# Single-cell genomics, metagenomics, and transcriptomics of *Rhizophydium megarrhizum*, an obligate fungal parasite of *Planktothrix agardhii*

**DOI:** 10.1007/s10452-026-10329-8

**Published:** 2026-07-22

**Authors:** Katelyn McKindles, Kensuke Seto, Steven Ahrendt, Asaf Salamov, Mansi Chovatia, Mei Wang, Kerrie Barry, Igor V. Grigoriev, R. Michael McKay, Timothy Y. James

**Affiliations:** 1 Department of Biology, Baylor University, Waco, TX USA; 2 Center for Reservoir and Aquatic Systems Research, Baylor University, Waco, TX USA; 3 Institute for Multidisciplinary Sciences (IMS), Yokohama National University, Yokohama, Kanagawa Japan; 4 U.S. Department of Energy Joint Genome Institute, Lawrence Berkeley National Laboratory, Berkeley, CA USA; 5 Department of Plant and Microbial Biology, University of California, Berkeley, CA USA; 6 Great Lakes Institute for Environmental Research, University of Windsor, Windsor, ON Canada; 7 Center for Great Lakes and Watershed Studies, Bowling Green State University, Bowling Green, OH USA; 8 Department of Ecology and Evolutionary Biology, University of Michigan, Ann Arbor, MI USA

**Keywords:** Chytridiomycota, Rhizophydiales, Planktothrix agardhii, Comparative genomics, Host-parasite interactions, Transcriptomics

## Abstract

Chytrids (phylum *Chytridiomycota*) are zoosporic fungi that play key roles as parasites of aquatic microorganisms, yet they are understudied and genomic resources for algal-infecting chytrids remain scarce. Here, we present the first comparative genomic analysis of multiple isolates of a single chytrid species (order *Rhizophydiales*) infecting the cyanobacterium *Planktothrix agardhii*. Isolates were collected from Sandusky Bay, Lake Erie, across two bloom years (2018 and 2019). Using single cell sequencing and metagenomic assembly, we generated individual genomes averaging 15.36 ± 0.12 Mbp in size with ~ 75% completeness, and a pangenome. Gene ontology analyses highlighted the presence of categories related to cellular structure, biosynthetic regulation, and interspecies interactions. As a preliminary exploration of gene expression during infection, we also performed RNA sequencing on a subset of size-sorted samples. These data suggest that chytrids consistently express high levels of cytoskeletal genes, alongside numerous hypothetical proteins, and that zoospores may upregulate carbohydrate-binding proteins implicated in host recognition. On the host side, *P. agardhii* showed transcriptional shifts in pathways associated with buoyancy and nutrient acquisition, patterns that could represent defensive adjustments or parasite-driven manipulation. Together, this study generates reference genomes for *Planktothrix*-infective chytrids, identifies conserved gene content across isolates from different bloom years, and provides preliminary transcriptomic insights into parasite and host responses. These resources lay the foundation for deeper investigations into chytrid genome evolution, infection biology, and their ecological roles in shaping cyanobacterial bloom dynamics.

**Supplementary Information:**

The online version contains supplementary material available at https://doi.org/10.1007/s10452-026-10329-8.

## Introduction

Chytrids (phylum *Chytridiomycota*) are zoosporic fungi that play diverse and ecologically significant roles in aquatic ecosystems (Gleason et al. 2008; Grossart et al. 2019). Unlike most other fungi, chytrids possess motile zoospores equipped with flagella, which enables them to actively seek out hosts or substrates (Sparrow 1960). Their unique life cycle, which includes transitions between zoospores and sporangia, allows chytrids to exploit a range of ecological niches (Gleason et al. 2017). Among their most important roles is parasitism, as chytrids infect phytoplankton (Frenken et al. 2017a) and even amphibians (Longcore et al. 1999), thereby shaping population dynamics, community structure, and ecosystem processes.

Many bloom-forming algae and cyanobacteria are susceptible to chytrid infection, which can influence bloom persistence and alter nutrient cycling (Sime-Ngando 2012). *Planktothrix agardhii*, a filamentous cyanobacterium common in eutrophic lakes, is notable for forming harmful algal blooms (HABs) that produce toxins such as microcystins (Christiansen et al. 2003; Kurmayer et al. 2015). Chytrid infections of *P. agardhii* can reduce bloom biomass (Sønstebø and Rohrlack 2011; Rohrlack et al. 2015; Wierenga et al. 2022) and redirect primary production through the “mycoloop,” a pathway in which chytrid zoospores provide a high-quality food source for grazers (Agha et al. 2016; Frenken et al. 2020; Rasconi et al. 2020). Despite these important ecological consequences, little is known about the genetics of chytrids and the molecular basis of chytrid-cyanobacterium interactions. Although the genetics and genomes of saprotrophic chytrids have been increasingly characterized (Amses et al. 2022), much less is known about parasitic chytrids, as most are obligate parasites that cannot be easily maintained in culture. Recent work has begun to address this gap by characterizing chytrid diversity and host specificity in cyanobacterial systems of the Laurentian Great Lakes. Multiple isolates of *Rhizophydium megarrhizum* (order *Rhizophydiales*) have been collected from *P. agardhii* blooms in Sandusky Bay, Lake Erie showing subtle differences in host range (McKindles et al. 2021) and internal transcribed spacer (ITS) sequences (McKindles et al. 2023).

Advances in fungal genomics and transcriptomics provide new opportunities to address these gaps. The expansion of genomic resources through platforms such as MycoCosm (Grigoriev et al. 2014) has shed light on chytrid genome architecture and have facilitated investigation into their unique cellular features. Transcriptome data in the model chytrid *Rhizoclosmatium globosum* revealed a major shift in gene expression across the zoospore to germling transition, with zoospores showing reduced metabolic activity but high investment in dispersal-related functions, while germlings upregulate translation, biosynthesis, and pathways needed for rhizoid growth and infection (Laundon et al. 2022). Similar patterns have been observed in *Batrachochytrium dendrobatidis* zoospores (Rosenblum et al. 2008) and during conidial germination in dikaryan fungi (Sharma et al. 2016), suggesting that transcriptional reprogramming during the dispersal-to-growth transition is a conserved feature across the fungal kingdom. It has also been proposed that chytrids exhibit evidence of plasticity, often mediated by repeats and mobile elements as observed in dikaryan fungi (van de Vossenberg et al. 2019). Despite these advances, genetic and genomic analyses of Chytridiomycota remain in their early stages, and much of their molecular diversity and evolutionary potential is still unexplored.

In this study, we present the first comparative genomic and transcriptomic framework for chytrids infecting *Planktothrix agardhii*. Using a combination of single-cell sequencing and metagenomic approaches, we assembled reference genomes for multiple *R. megarrhizum* isolates collected from Sandusky Bay. We then used RNA sequencing to examine both parasite gene expression across infection stages and the corresponding transcriptional response of *P. agardhii*. We hypothesized that chytrid genome sequencing would reveal signatures of their obligate parasitic lifestyle, including enrichment of genes related to cytoskeletal remodeling as part of lifecycle transitions (zoospore to sporangium), host recognition, and interactions with other organisms. We further hypothesized that transcriptomic analyses would capture dynamic shifts in both chytrid and host gene expression during infection, highlighting candidate molecular mechanisms of invasion and defense. By testing these hypotheses, our study establishes foundational genomic resources for algal chytrids and provides a framework for exploring the genetic basis of chytrid functional diversity and parasitism.

## Materials and methods

### Host and parasite culture maintenance

After isolation (described in McKindles et al. 2021), both the host cyanobacteria isolates (*Planktothrix agardhii* 1031 and 1803) and obligate chytrid parasite isolates (*Rhizophydium megarrhizum* C01, C02, C03, C06, C07, C08, C10, C21, C22, C23, and C24) were maintained as non-axenic batch cultures. Cultures were grown on Jaworski’s medium (JM; Jaworski et al. 1981) at 20 °C in lighted incubators that were set to a light-dark cycle of 12 h:12 h and 25 μmol photons m^−2^ s^−1^. Infections were monitored via microscope observation every other day for up to two weeks, at which point the host culture was severely infected (i.e., the percent infected filaments with visible sporangia were above 70%), and infections were then diluted 1:100 in fresh late-log phase host culture.

### Single cell isolation for *Rhizophydium megarrhizum* C02 whole genome sequencing (PlkC2_A and PlkC2_B)

Since the chytrid was unable to be grown as an independent culture, isolation of single infected filament containing a mature sporangium was carried out to minimize contamination with other organisms as previously described, with modifications (Seto et al. 2023). In brief, *P. agardhii* 1031 infected with *R. megarrhizum* C02 was screened using a Nikon TMS Inverted microscope (Nikon, Tokyo, Japan). Infected filament fragments that housed mature but still full sporangia were targeted. These infected filament fragments were isolated manually using 10 µL extended pipette tips. The isolated material was washed by serial transfer in small drops of sterilized media for at least 5 transfers. Finally, the infected filament fragments were transferred into 200 µL PCR tubes containing a total volume of 1– 2 µL of UV-sterilized water and kept at −80 °C until the DNA was extracted.

Whole genome amplification was performed as described in Seto et al. (2023). In brief, DNA extraction and whole genome amplification of isolated cells was conducted by multiple displacement amplification (MDA), using half reactions of the Qiagen REPLI-g Single Cell Kit (Qiagen, Germantown, MD, USA) following the protocol established by Davis et al. (2019). The DNA concentrations of the MDA products were measured with the Qubit 4 Fluorometer (Thermo Fisher Scientific, Waltham, MA, USA).

Two biological replicate samples (PlkC2_A and PlkC2_B) were sent to the Joint Genome Institute (JGI) for whole genome sequencing and annotation according to the JGI annotation pipeline as part of the MycoCosm project (Grigoriev et al. 2014). Both genomes were sequenced with Illumina. Plate-based DNA library preparation for Illumina sequencing was performed on the PerkinElmer Sciclone NGS robotic liquid handling system. 1 ng of DNA was fragmented and adapter ligated using the Nextera XT kit (Illumina) and unique 8 bp dual-index adapters (IDT, custom design). The ligated DNA fragments were enriched with 12 cycles of PCR and purified using Coastal Genomics Ranger high throughput agarose gel electrophoresis size selection to 450–600 bp. The prepared libraries were then quantified using KAPA Illumina library quantification kit (Roche) and run on a LightCycler 480 real-time PCR instrument (Roche). The quantified libraries were then multiplexed, and the pool of libraries was then prepared for sequencing on the Illumina NovaSeq 6000 sequencing platform using NovaSeq XP v1.5 reagent kits (Illumina), S4 flow cell, following a 2 × 150 indexed run recipe. The sequenced reads filtered and trimmed for quality were assembled using SPAdes (Bankevich et al. 2012). First, sequence similarity searches were performed for every contig using MMseqs2 (Steinegger and Söding 2017) against the NCBI non-redundant (NR) database and all proteins from JGI MycoCosm (Grigoriev et al. 2014). For each contig, the taxonomic unit (species or genus, or in some low-resolution cases, a higher rank such as Fungi) was selected with the highest representation. These contigs were grouped into bins corresponding to that taxonomic unit. Because many contigs lacked significant homology to any known sequences, the bins were further refined by running MetaBAT2 (Kang et al. 2019) on each contig. Only those MetaBAT2 bins for which the majority of contigs were assigned to the selected taxonomic unit were retained, and contigs without detectable homology were appended to these bins. For PlkC2_A, the majority of contigs showed homology to members of the Globomycetaceae family (order Rhizophydiales), and this taxonomic unit was selected for bin assignment. In contrast, for PlkC2_B, taxonomic resolution was lower, and the contigs were assigned to Fungi incertae sedis, representing early-diverging fungal lineages. In the first step, all contigs assigned to Globomycetaceae (PlkC2_A) and Fungi incertae sedis (PlkC2_B) were grouped into two initial bins. In the second step, additional contigs were incorporated from MetaBAT2-generated bins in which the majority of contigs were assigned to the same respective taxonomic units, Globomycetaceae for PlkC2_A and Fungi incertae sedis for PlkC2_B. Subsequent taxonomic refinement was performed after genome annotation using the JGI Annotation pipeline (Grigoriev et al. 2014) where higher-resolution assignments were obtained based on whole-genome phylogenetic reconstruction.

Both resulting genomes (*R. megarrhizum* PlkC2_A and *R. megarrhizum* PlkC2_B) were imported into Geneious Prime v.2024.0.5 to undergo whole genome alignment comparison using Mauve v2015-02-25 (Darling et al. 2004). First, the contigs of *R. megarrhizum* PlkC2_B were reordered according to *R. megarrhizum* PlkC2_A using the MCM algorithm, then both whole genomes were aligned using the progressiveMauve algorithm. Seed weight and minimum Locally Collinear Blocks (LCB) score were automatically calculated, and the program was set to not assume collinear genomes and do a full alignment. To assess completeness against each other, each genome was mapped to the other using QUAST (Gurevich et al. 2013). General genome completeness was assessed using gVolante (Nishimura et al. 2017), which utilizes BUSCO_v5 (Simão et al. 2015; Manni et al. 2021) database with Fungi selected ortholog set.

### Metagenome assembled *Rhizophydium megarrhizum* genomes

While *R. megarrhizum* C02 has been characterized over several previous publications (McKindles et al. 2021, 2023; Wagner et al. 2023), additional strains were isolated in 2018 (isolates C01, C03, C06, C07, C08, and C10) and 4 more in 2019 (isolates C21, C22, C23, and C24). Each isolate was grown on *P. agardhii* 1803 and collected on 0.22 µm PES Sterivex cartridge filters (MilliporeSigma, Burlington, MA, USA) for metagenomic analysis. The DNA from the infected cultures was extracted using the Qiagen DNeasy PowerWater Sterivex kit following the manufacturer’s instructions. The DNA was checked for concentration and quality using a Nanodrop Spectrophotometer (Thermo Fisher Scientific, Waltham, MA, USA), then was sent to Discovery Life Sciences (Huntsville, AL), where the samples were sequenced on a NovaSeq 6000.

Metagenome-assembled genomes (MAGs) were generated by de novo alignment followed by binning and Kraken2 identification of fungal bins. Initial MAG generation was run in OmicsBox v3.1.11. Raw reads were preprocessed using Trimmomatic v0.39 (Bolger et al. 2014), which removed adaptors, trimmed 15 bp off of the 5’ end, and filtered the reads based on average quality (25) and minimum length (36). After preprocessing, the reads were assessed with FastQC (Andrews 2010). Reads that passed trimming and QC underwent De Novo assembly via metaSPAdes v3.15.4 (Nurk et al. 2017) using OmicsBox default parameters: Automatic K-mer size (33 and 55). Contig files were exported from OmicsBox for binning using MetaBAT v2.15 (Kang et al. 2019) using default parameters; minContig 2500, minCV 1.0, minCVSum 1.0, maxP 95%, minS 60, maxEdges 200, minCLsSize 200,000, and random seed. The resulting bins were imported back into OmicsBox for taxonomic classification using Kraken v2.1.3 (Wood and Salzberg 2014; Wood et al. 2019), using the RefSeq 2024.04 database, confidence filter set to 0.05, and minimum hit groups of 2. Fungal bins were identified with the classification “Eukaryota: Chytridiomycota: Rhizophydiales”. Occasionally, *Bacteria* contigs were included in the fungal bins, which were manually confirmed via NCBI Blast search (standard databases, megablast) and removed from the fungal bin. MAG completeness was assessed using gVolante (Nishimura et al. 2017), which utilizes BUSCO_v5 (Simão et al. 2015) database with Fungi selected ortholog set.

MAG chytrid genomes were imported into Geneious Prime v.2024.0.5 to undergo whole genome alignment comparison using Mauve v2015-02-25 (Darling et al. 2004). As *R. megarrhizum* C06 was the most complete using BUSCOs scores, it was chosen as the reference genome for contig reordering. Contigs were reordered using the MCM algorithm, automatically calculated seed weight and automatically calculated minimum Locally Collinear Block (LCB) score. Ordered contigs were saved. After ordering, all the chytrid genomes were aligned using the progressive Mauve algorithm with the same default parameters as the contig reordering.

Annotation of the fungal MAGs was performed in OmicsBox v3.2.9. MAG files underwent repeat masking using RepeatMasker v4.0.9 (Smit et al. 2013), which utilizes HMMER search engine and the following configurations: species designation “4751 Fungi”, soft masking, and identification of both “Interspersed repeats” and “Simple repeats and low complexity DNA.” Output softmasked fasta files were then used for eukaryotic gene finding based on the AUGUSTUS v3.5.0 software (Hoff and Stanke 2019). Closest species designation was set to Fungi – *Ascomycota* – *Saccharomyces cerevisiae* (RM11-1a_1). Note that there was a *Chytridiomycota* choice (*Chytridiomycota*-*Monoblepharidomycetes*-*Gonapodya prolifera*), but gene model output was higher with the better-quality reference (i.e. 207 genes identified with *Gonapodya prolifera* reference versus 9,233 genes identified with *Saccharomyces cerevisiae* reference when using *R. megarrhizum* PlkC2_A as a test genome). Other parameters include predicting on “both strands,” allowed “partial” gene structure, and output “introns,start,stop,” with all other parameters kept as default. Gene finding mode was set to Ab initio prediction.

Predicted genes and proteins were annotated using eggNOG-Mapper v2.1.0 with EggNOG v5.0.2 (Huerta-Cepas et al. 2019) and InterProScan 5 (Jones et al. 2014). InterProScan used the public EMBL-EBI InterPro web-service to search for families, domains, sites, and repeats using NCBIfam, SFLD, Panther, HAMAP, ProSiteProfiles, SMART, CDD, PRINTS, PfamA, and PIRSF databases. eggNOG-Mapper searched for all target orthologs and used non-electronic GO evidence. Both sets of functional annotations were merged with the cds file (merged EggNOG 5 GO annotations had seed ortholog e-value set to 1E-3 and bit-score set to 60), then validated based on True-Path-Rule for removing redundant GO terms via OmicsBox GO Annotation functions.

General Feature Format (GFF) files containing GO annotations were then exported from OmicsBox using the Export GFF function. Coordinate sequence files were exported as GFF 3 files and included transcript, CDS, and gene features. “Add GOs from project” was checked, which was derived from the merged cds file. The GOs were added to the transcript features, based on the parent attribute name. Both the GFF files and the contig fasta files were imported to Kbase (Arkin et al. 2018) for additional analyses.

### Comparative genomics between *Rhizophydium megarrhizum* isolates

Aiming to assess the level of discrepancy among the genomes, whole genome sequence identity was computed using FastANI v0.1.3 (Jain et al. 2018) in Kbase (Arkin et al. 2018). Both MAG and single-cell genome sequences were imported and analyzed. The ANI was recorded as the lower of the two pairwise estimates and the alignment percentage was calculated as the number of matches divided by the total potential matches for this same pair.

Two *R. megarrhizum* pangenomes were generated using OrthoMCL v.2.0 (Li et al. 2003). The first one included *R. megarrhizum* PlkC2_A from the single-cell genome set, and 10 of the 11 *R. megarrhizum* MAGs, leaving out the two low completeness genomes (*R. megarrhizum* PlkC2_B and *R. megarrhizum* MAG C08). The second only analyzed the 10 *R. megarrhizum* MAGs. In both instances, OrthoMCL was run using default parameters: num-descriptions -100,000, num_alignments -100,000, e-value -1e-5, word_size -3, gapopen -11, gapextend -1, matrix -BLOSUM62, threshold -11, comp_based_stats -2 (Composition-based score adjustments as in Bioinformatics 21:902-911, 2005, conditioned on sequence properties), seg -yes, lcase_masking -false, xdrop_gap_final -25, window_size -40, use_sw_tback -false, mcl_p -10,000, mcl_s -1100, mcl_r -1400, and mcl_pct -90. The pangenome breakdown is provided in Table S1. From the pangenome file, a pangenome circle plot was generated using Kbase’s Pangenome Circle Plot v1.6.0, which is based on Pangenome Calculator OrthoMCL (Fischer et al. 2011). For the pangenome that contains the single-cell genome *R. megarrhizum* PlkC2_A, *R. megarrhizum* PlkC2_A was chosen as the base genome to order the ortholog clusters. For the *R. megarrhizum* MAGs pangenome, as *R. megarrhizum* C06 was the most complete using BUSCOs scores, it was chosen as the base genome to order the ortholog clusters.

### Transcriptome during C02 chytrid infection on *Planktothrix agardhii* 1031

During routine maintenance of the obligate chytrid parasite on host *P. agardhii* 1031, four samples were collected for metatranscriptomic analysis: the uninfected host, a sample of the infected host displaying the ball aggregate phenotype as described in McKindles et al. (2021), an infected sample concentrated on a 11 µm nylon filter (> 11 µm fraction), and the filtrate from that same sample (< 11 µm fraction). In brief, subsamples of infected material were collected onto 0.22 µm PES Sterivex cartridge filters (MilliporeSigma) and stored at −80 °C until extracted. Total RNA was extracted using the Qiagen RNeasy PowerWater Kit (Qiagen, Germantown, MD, USA) by first removing the filter from the cartridge under sterile conditions in a laminar flow hood, then following the manufacturer’s instructions with the following notes: 60 µL of Qiagen RNase Free DNase solution was incubated for 30 min at room temperature to elute the RNA from the extraction column. Remaining DNA was removed using the Ambion Turbo DNA-free kit (Thermo Fisher Scientific) according to manufacturer’s instructions. The RNA was quantified using the Nano Drop 2000 (Thermo Fisher Scientific, Waltham, MA, USA).

Extracted RNA was sent to Discovery Life Sciences (Huntsville, AL), where the samples were treated to reduce rRNA using TruSeq Stranded Total RNA with Ribo-Zero Plant kit (Illumina, San Diego, CA). RNA was sequenced on NovaSeq 6000 with paired-end reads of 150 base pairs.

The metatranscriptome reads were analyzed using OmicsBox software v3.4.6 using the transcript-level analysis workflow. The paired end reads were preprocessed using Trimmomatic v0.39 (Bolger et al. 2014), which removed adaptors, trimmed 15 bp off the 5’ end, and filtered the reads based on average quality (25) and minimum length (36). After preprocessing, the reads were assessed with FastQC (Andrews 2010). The trimmed reads underwent taxonomic classification using Kraken2 v.2.1.3 (Wood et al. 2019), which uses the Kraken2 RefSeq 2024-11 WGS database and filters based on confidence at 0.05 (out of 1), and 2 minimum hits per group to be counted. Kraken2 was also run using the UNITE 10.0 ITS database with the same confidence filters.

Reads that passed trimming and QC underwent RNA-seq Read Quantification, which uses the RSEM v1.3.3 software package (Li and Dewey 2011) and Bowtie2 v2.5.3 (Langmead and Salzberg 2012) to estimate gene expression using a set of reference transcript sequences. The program was set to estimate a read start position distribution (RSPD) and map the reads in a non-strand specific manner. Transcripts were mapped to either the *P. agardhii* no.976 RefSeq genome (GCF_904830935.1) or the predicted genes from the *R. megarrhizum* PlkC2_A single-cell genome. The raw counts tables from the transcript mapping were normalized as the Trimmed mean of M value (TMM). The normalized read count tables were exported into Rstudio v2023.06.1 + 524 for the generation of figures. Heatmaps of the top 20 expressed *P. agardhii* genes and top 25 expressed *R. megarrhizum* genes were generated using the ggplot2 package.

Because the Large (> 11 µm) fraction sample and the uninfected control sample were the two samples that had the largest portion of reads that could be mapped to the *P. agardhii* 1031 genome, they were chosen for Pairwise Differential Expression Analysis (Without Replicates) which is based on the Bioconductor v3.10 package NOISeq v2.30.0 (Tarazona et al. 2015), which compares samples by simulating replicates. Genes with low counts (defined as less than 1.0 CPM) were filtered out. Replicate simulation was set to generate 5 simulated replicates, using 0.2 the size of the sample library to run the simulation, and include 0.02 variability of total number of reads. These same parameters were used to run the differential expression of *R. megarrhizum* between the Small (< 11 µm) and the Large (> 11 µm) fraction. In both instances, the data was exported into Rstudio v2023.06.1 + 524 for the generation of expression plots using the ggplot2 package.

## Results

### *Rhizophydium megarrhizum* C02 single cell genome assembly

Two single cell samples from culture C02 were used for whole genome sequencing. *R. megarrhizum* PlkC2_A yielded a 15.32 Mbp genome assembly, with 1,257 contigs (scaffold N50 of 206) and 8,572 gene models. *R. megarrhizum* PlkC2_B yielded a 11.51 Mbp genome assembly, with 1,688 contigs (scaffold N50 of 319) and 6,479 gene models. When mapped against each other, PlkC2_A includes 84.25% of PlkC2_B, indicating that 15.76% of PlkC2_B’s genome is not included in PlkC2_A (Fig. S1). BUSCO analysis shows that PlkC2_A is 75.4% complete (S:74.7%, D:0.7%) and PlkC2_B is 52.0% complete (S:51.6%, D:0.4%). BUSCO analysis also showed that at the protein level, PlkC2_A was 77.7% complete.

### *Rhizophydium megarrhizum* isolate metagenome assembled genomes (MAGs)

Eleven isolate *R. megarrhizum* MAGs were generated (Table 1). Except for *R. megarrhizum* C08, which is much less complete than the others, all isolates had an assembly size of 15.36 ± 0.12 Mbp broken into 1279.3 ± 36.83 contigs with a completeness score of 75.78 ± 0.62%. The MAGs yielded similar values to the *R. megarrhizum* C02 single cell genome assembly, except that they contained on average 1,317 ± 61 more gene models than the single cell genome, possibly due to the allowed partial gene structure option in AUGUSTUS. This was confirmed through BUSCO analysis on MAG C06, as the completeness at a genome level was 76.9% (Table 1), but at a protein level, the completeness was only 40.5%, as BUSCO does not count fragmented genes.

**Table 1:** Description of 11 *R. megarrhizum* Metagenome assembled genomes (MAGs)

Isolate designation	Size of assembly (Mbp)	Number of contigs	N50 seq length (nt)	Number of genes	Complete BUSCOs (%)
C01	15.21	1379	16,776	9,845	C:74.6%[S:73.1%,D:1.5%],F:4.9%,M:20.5%,n:758
C02	15.56	1277	18,921	9,988	C:75.9%[S:75.1%,D:0.8%],F:5.0%,M:19.1%,n:758
C03	15.16	1261	18,453	9,803	C:76.3%[S:75.6%,D:0.7%],F:4.6%,M:19.1%,n:758
C06	15.29	1257	19,150	9,852	C:76.9%[S:76.1%,D:0.8%],F:4.0%,M:19.1%,n:758
C07	15.41	1278	18,797	9,916	C:75.9%[S:75.1%,D:0.8%],F:4.6%,M:19.5%,n:758
C08	4.89	1273	3763	3,392	C:20.5%[S:20.4%,D:0.1%],F:7.4%,M:72.1%,n:758
C10	15.36	1246	19,436	9,869	C:75.2%[S:74.4%,D:0.8%],F:4.5%,M:20.3%,n:758
C21	15.40	1254	19,227	9,913	C:75.9%[S:74.7%,D:1.2%],F:4.5%,M:19.6%,n:758
C22	15.43	1301	18,507	9,929	C:75.1%[S:74.3%,D:0.8%],F:4.6%,M:20.3%,n:758
C23	15.26	1256	19,373	9,819	C:76.0%[S:74.5%,D:1.5%],F:4.0%,M:20.0%,n:758
C24	15.47	1284	18,596	9,959	C:76.0%[S:75.1%,D:0.9%],F:4.9%,M:19.1%,n:758

**Fig. 1:**
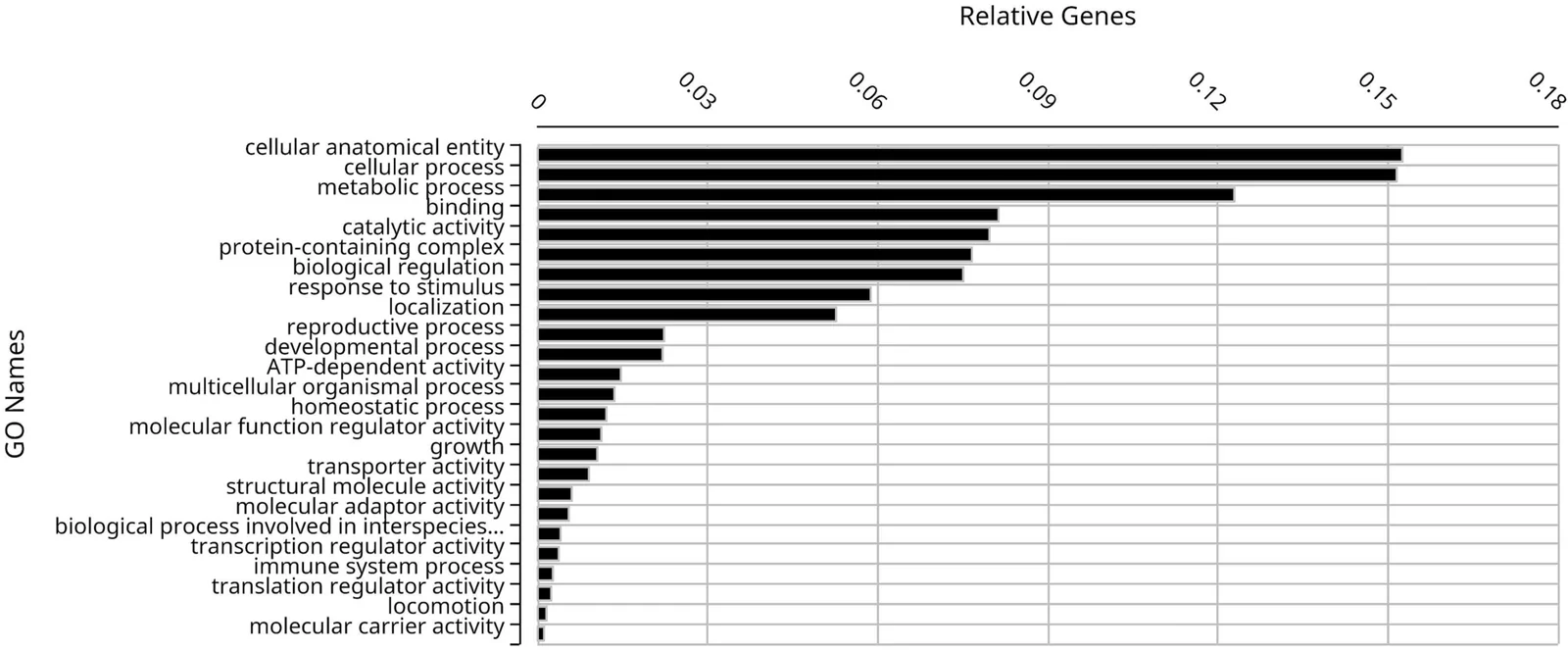
Relative abundance of top GO annotations (cellular components, biological processes, and molecular functions) in *R. megarrhizum* MAGs

The gene annotations for the *R. megarrhizum* MAGs were similar across all isolate genomes. The most abundant GO annotations (Fig. 1) were related to cellular processes such as biological regulation, localization, reproduction, and development, and metabolic processes such as catalytic activity, response to stimuli, and protein-containing complexes. Other GO annotations of note included biological processes involved in interspecies interactions and locomotion.

### Comparative genomics between *Rhizophydium megarrhizum* isolates

Given the similar values between *R. megarrhizum* single cell isolate PlkC2_A and the *R. megarrhizum* MAGs, further characterization was needed to understand their level of relatedness, including average nucleotide identity (ANI) and alignment percentage (AP) (Fig. S2). Because of the reduced completeness in *R. megarrhizum* single cell isolate PlkC2_B and *R. megarrhizum* MAG C08, both had reduced ANI and AP compared to the other genomes. Across the remaining genomes, ANI was greater than 99% (Fig. S2a). AP varied more than ANI (Fig. S2b), where *R. megarrhizum* single cell isolate PlkC2_A, *R. megarrhizum* MAG C01 and *R. megarrhizum* MAG C24 had an average AP of 85.9, 88.6, and 89.5% respectively. The rest of the MAGs aligned with each other consistently around 92.5 ± 0.003%. The closest AP could be found between *R. megarrhizum* MAG C03, *R. megarrhizum* MAG C06, and *R. megarrhizum* MAG C07, with an average AP of 93.3 ± 0.003% between the three genomes.

**Fig. 2:**
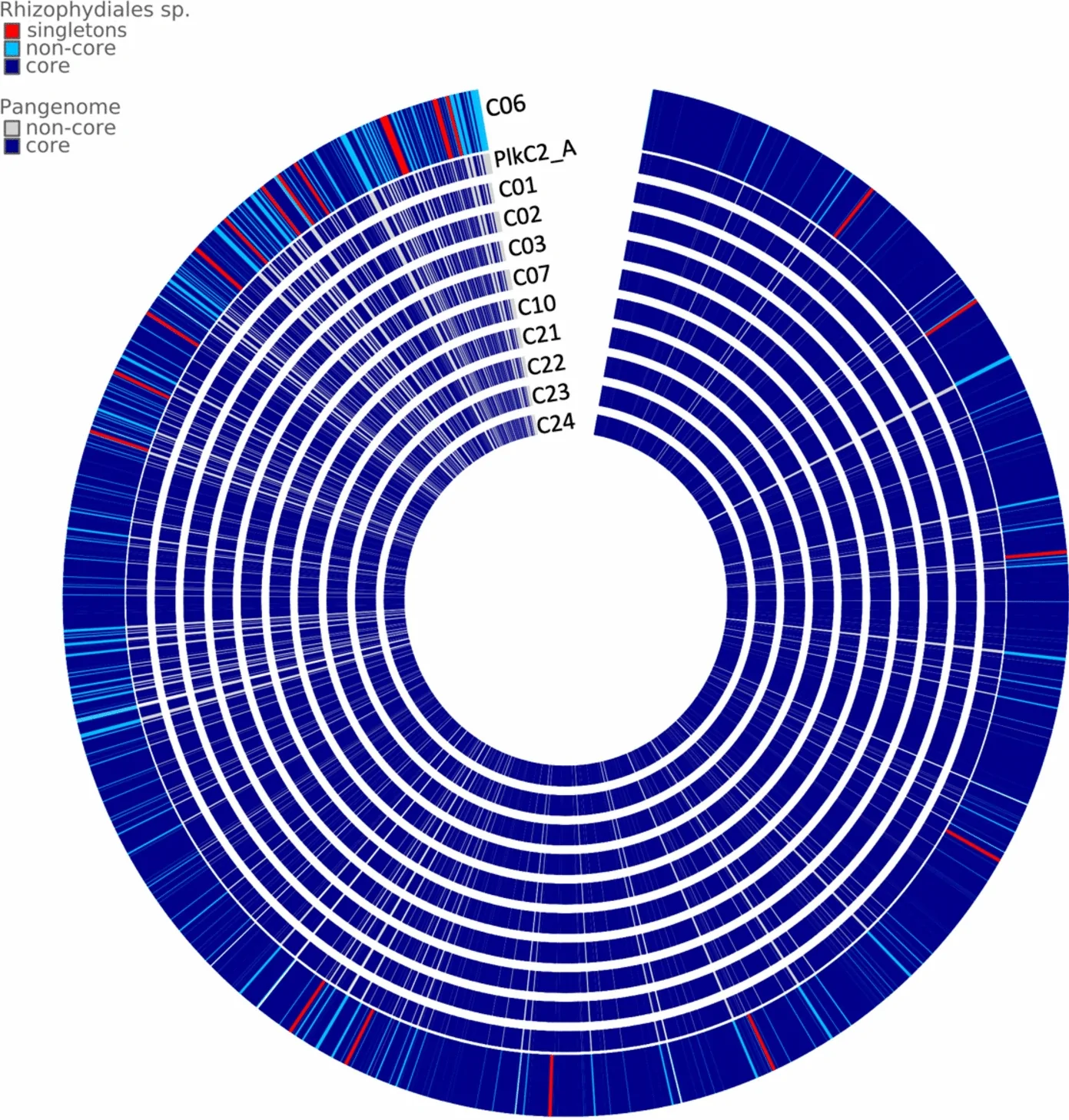
Pangenome of *R. megarrhizum* isolates. In the generation of the pangenome, *R. megarrhizum* MAG C08 and *R. megarrhizum* single-cell isolate PlkC2_B were dropped due to genome incompleteness. The pangenome is classified as core (ortholog sets with at least one gene from the ortholog set with a gene in each of the genomes), non-core (sets that do not contain a gene in each of the genomes), and singletons (genes with no sequence homology to genes in any other genomes)

After the removal of *R. megarrhizum* single cell isolate PlkC2_B and *R. megarrhizum* MAG C08, the remaining related genomes were then used to generate a *R. megarrhizum* pangenome (Fig. 2). The pangenome analysis identified 17,017 genes with translation, 15,756 of which are in homolog families and 1,261 of which are found only as a singleton in one genome (Table S2). Many singleton genes were either unannotated (35.6%) or identified as hypothetical proteins (39.5%). Among the annotated singleton genes, recurring functional categories included ankyrin repeat-containing domain proteins (1.2%), protein kinase-like domain-containing proteins (0.6%), and histone-fold-containing proteins (0.4%), suggesting that while the majority of singleton genes were unannotated or hypothetical, some isolate-specific variability may involve regulatory or protein interaction-associated functions. The *R. megarrhizum* MAGs were more conserved between the genomes, with an average of 95.7 ± 0.06% of the genes found in homolog groups and only 4.3 ± 0.06% genes found in a single genome. On the other hand, the *R. megarrhizum* single cell isolate PlkC2_A had only 72.4% of its genes in homolog groups, indicating that 27.6% of the gene models were only found in the single cell isolate. This further indicates that while the *R. megarrhizum* MAGs and the *R. megarrhizum* single cell isolate PlkC2_A have similar genome lengths, ANI, and AP, discrepancies in predicted gene content are likely driven primarily by differences in gene prediction workflows, particularly the retention of partial gene models in the MAG assemblies.

The pangenome including *R. megarrhizum* single-cell isolate PlkC2_A and the 10 *Rhizophydium* MAGs (Fig. 2) identified 11,074 ortholog families and their associated function, of which the top 15 are listed here (Table 2). The cluster with the highest protein coding gene count (Cluster1), as well as several other high-count clusters (Cluster7, Cluster10, Cluster14, and Cluster15) all encode for P-loop containing nucleoside triphosphate hydrolase proteins. Other notable functions that appear to be highly conserved across the genomes include a Dynein motor protein (Cluster2) and a Chitin synthase-domain containing protein (Cluster6). As is common in many genomes, and fungal genomes in particular, some ortholog clusters encode for hypothetical proteins (Cluster8, Cluster12, and Cluster13).

**Table 2:** Top pangenome ortholog groups between *R. megarrhizum* single-cell isolate PlkC2_A and the 10 *R. megarrhizum* MAGs. Complete dataset is available in Supplementary Table S2

Family	Function	Protein coding gene count	Genome count
cluster1	P-loop containing nucleoside triphosphate hydrolase protein	292	11
cluster2	Dynein heavy chain and region D6 of dynein motor-domain containing protein	175	11
cluster3	ankyrin repeat-containing domain protein	116	11
cluster4	ClpP/crotonase-like domain-containing protein	113	11
cluster5	ClpP/crotonase-like domain-containing protein	111	11
cluster6	Chitin synthase-domain containing protein	67	11
cluster7	P-loop containing nucleoside triphosphate hydrolase protein	62	11
cluster8	hypothetical protein	59	11
cluster9	G8 domain-domain containing protein	59	11
cluster10	P-loop containing nucleoside triphosphate hydrolase protein	56	11
cluster11	Sec63 Brl domain-domain containing protein	53	11
cluster12	hypothetical protein	47	11
cluster13	hypothetical protein	42	11
cluster14	P-loop containing nucleoside triphosphate hydrolase protein	42	11
cluster15	P-loop containing nucleoside triphosphate hydrolase protein	42	11

### Metatranscriptome of *Rhizophydium megarrhizum *infection on *Planktothrix agardhii*

Three individual chytrid infected samples and an uninfected control sample underwent transcriptome analysis to identify fungal genes that were involved in the infection process. The first was during a late stage chytrid infection, in which *P. agardhii* 1031 was showcasing the ball defense aggregate morphology (Fig. S3). This aggregate morphology has been previously observed in *Planktothrix* cultures during chytrid infection (McKindles et al. 2021), where elongated filaments wrap around one another into dense spherical aggregates that may reduce zoospore access to susceptible filament surfaces. The other two samples were size fractionated during the infection process to include a greater than 11 µm pore sized fraction, and a smaller than 11 µm pore sized fraction. Taxonomically (Fig. S4), the uninoculated control was 86.77% *Microcoleaceae* (*Cyanobacteria)*, while the infected samples ranged from 9.02 to 82.89% *Cyanobacteria*. In addition, the infected samples were 0.015–0.37% *Chytridiomycota* (Fig. S4, collapsed in “Other”). A considerable portion of the reads were unclassified in the smaller than 11 µm pore sized fraction and the ball defense aggregate morphology sample (Fig. S4). If analyzed using the UNITE database for ITS sequences, *Chytridiomycota* increased to 0.05–1.54% of the total reads (data not shown), meaning 0.05–1.54% of total reads were rDNA of chytrids and represent a small percentage of the total chytrid reads.

The ball defense sample had 3.58% of reads mapped to *P. agardhii* 1031 (host) and 0.08% of reads mapped *R. megarrhizum* single-cell isolate PlkC2_A (chytrid). The greater than 11 µm pore sized fraction had 38.41% of reads mapped to the host and 2.22% of reads mapped to the chytrid, while the smaller than 11 µm pore sized fraction had 11.5% of reads mapped to the host and 1.56% of reads mapped to the chytrid. The uninfected *P. agardhii* 1031 sample had a total of 35.8% of reads mapped to the RefSeq genome.

#### *Rhizophydium megarrhizum* C02

**Fig. 3:**
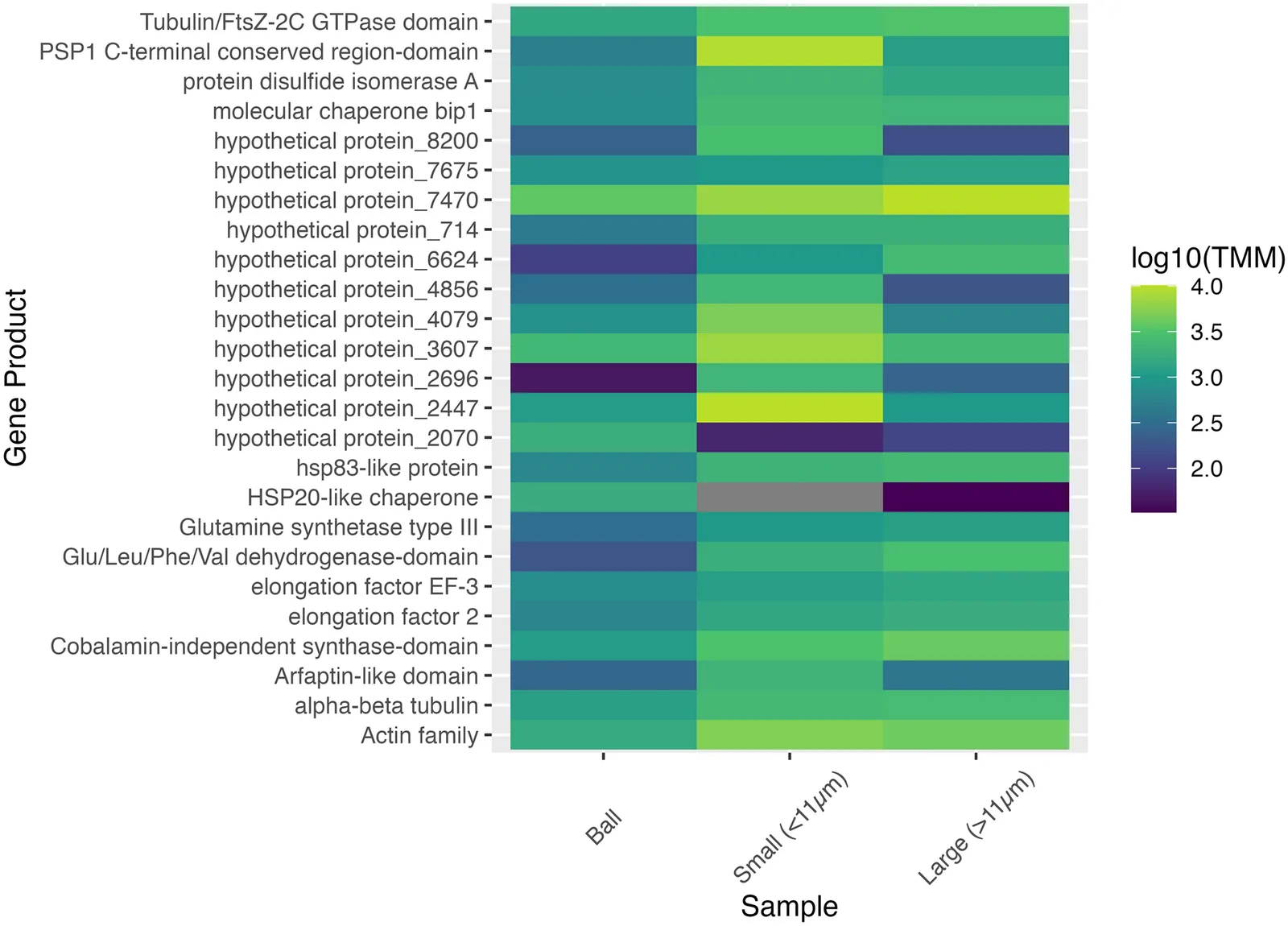
Heatmap of top 25 expressed *R. megarrhizum* genes during an infection on *P. agardhii*. Expression is displayed as log10 transformed trimmed mean of M (TMM) values

*R. megarrhizum* transcripts were observed for three infected samples (Fig. 3). Around half of the transcripts were annotated as hypothetical genes and all genes showed varied levels of expression across the three samples. Highly transcribed genes include cytoskeleton genes such as tubulin and actin, molecular chaperon genes such as hsp83 and hsp20, and biochemical processes. When looking closer at the differences between the small infective particles (zoospores) and large infective particles (sporangia), increased expression of carbohydrate-binding family 9-like protein (*CBD9*) and Concanavalin A-like lectin/glucanase domain-containing protein (*ConA*) suggest carbohydrate binding may be upregulated during the zoospore stage (Fig. S5).

#### *Planktothrix agardhii* 1031

**Fig. 4:**
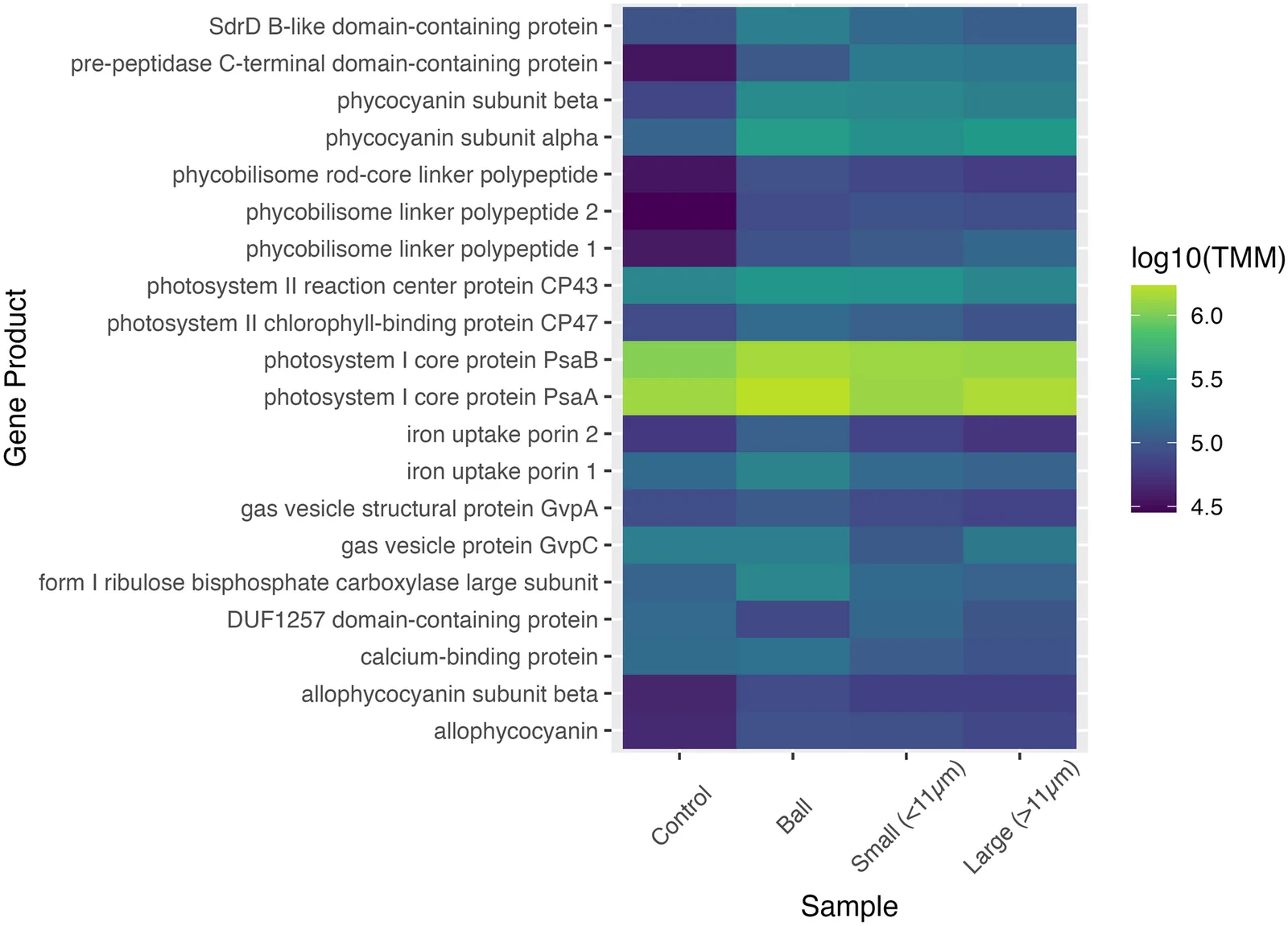
Heatmap of top 20 expressed *P. agardhii* genes, both uninfected (Control) and infected with *R. megarrhizum* (Ball, Small (< 11 µm), and Large (> 11 µm)). Expression is displayed as log10 transformed trimmed mean of M (TMM) values

*P. agardhii’*s most abundant transcripts are related to photosynthesis, regardless of infection state (Fig. 4). Several of these photosynthesis genes were more abundant in the chytrid infected samples than the uninfected control. In particular, the phycobilisome linker polypeptide genes are 2.93 ± 0.34 times higher than the control and the phycocyanin subunits alpha and beta are 2.86 ± 0.39 times higher than the control. Alternatively, the gas vesicle proteins (*GvpA* and *GvpC*) were on average 10% less abundant. If we compare the uninfected control to the greater than 11 µm pore sized fraction, there is a decrease in expression of nitrogen related genes in the chytrid infected sample (Fig. S6). These potentially downregulated genes include several nitrate ABC transporter genes such as *NrtA*, *NrtB*, and *NrtCD*, which were, on average, 147.68 ± 94.73 times more expressed in the control than in the infected sample.

## Discussion

This study presents the first comparative genomic analysis of multiple isolates of the same chytrid species infecting the cyanobacterium *Planktothrix agardhii*. All *Rhizophydium megarrhizum* isolates were collected from the same location (Sandusky Bay, Lake Erie, OH, USA), but across two different bloom years (2018 and 2019). Previous work has shown these isolates differ slightly, either in host preference (McKindles et al. 2021) or in their ITS sequences (McKindles et al. 2023). Here, our goal was to deepen our understanding of chytrid genetics and to begin establishing reference genomes to support future research on *Chytridiomycota*.

We used both single-cell sequencing and metagenomics to generate individual genomes and a pangenome for *R. megarrhizum* (Fig. 2). The individual genomes, assembled from both single-cell and metagenomic data (MAGs), were similar in size and structure, averaging 15.36 ± 0.12 Mbp across 1279.3 ± 36.83 contigs, with a mean completeness of 75.78 ± 0.62%. Assuming full completeness would require ~ 25% more sequence, these *R. megarrhizum* genomes are relatively small compared to other chytrid genomes published through the MycoCosm project (Grigoriev et al. 2014; Ahrendt et al. 2022), including the related species *Globomyces pollinis-pini* (Amses et al. 2022). However, most of these chytrid species with available genomes are culturable and saprotrophic. Additionally, many chytrid genomes may not reach full completeness because the fungi-odb10 database is prepared largely using dikaryotic fungi (Manni et al. 2021).

Despite originating from the same geographic location, the *R. megarrhizum* isolates displayed measurable genomic variation, including differences in ortholog group composition and ITS sequence structure identified here and in previous studies (McKindles et al. 2021, 2023). These patterns suggest that chytrid populations associated with cyanobacterial blooms may possess greater functional and evolutionary diversity than previously appreciated. Such variation could contribute to differences in host preference, infection dynamics, environmental tolerance, or resource acquisition strategies, although direct functional validation will be required to test these hypotheses. Comparative genomic analyses within parasitic chytrid species remain rare due to the limited availability of high-quality genomes, and chytrid taxa are often implicitly treated as genomically homogeneous. By generating multiple genomes from isolates of the same species, this study provides an initial framework for investigating intraspecific diversification and eco-evolutionary processes in chytrid–cyanobacteria interactions.

The most abundant GO terms across the genomes include common general terms such as cellular processes and metabolic processes, but also more specific terms such as response to stimuli, locomotion, and biological processes involved in interspecies interactions between organisms (Fig. 1). Cellular anatomical structure, which encompasses a large portion of the annotated genome, is related to organelles and membranes. This allocation towards cellular structure is likely a result in the distinctive life stages: as zoospores, chytrids lack a cell wall and increase the number of total organelles, including their ribosomes, rumposomes, mitochondria, which shifts to more other organelles such as golgi apparatus and endomembrane space as an immature thallus (Laundon et al. 2022). These stage transitions also likely come with an increased need for biosynthetic and genetic regulation processes, which fall under the cellular and metabolic processes GO terms, which are second and third in the GO terms chart, respectively (Fig. 1).

In addition to genome sequencing, a subset of samples was extracted for preliminary RNAseq analysis to examine chytrid gene expression during infection (Fig. 3) and the corresponding transcriptional response of the host, *P. agardhii* (Fig. 4). In infected samples, *P. agardhii* showed increased expression of photosynthesis-related genes, including those encoding components of the phycobilisome such as phycocyanin, a major light-harvesting pigment-protein complex (Zilinskas and Greenwald 1986; Singh et al. 2015). At the same time, genes associated with gas vesicle formation were downregulated (Fig. 4). Because gas vesicles regulate buoyancy in the water column (Walsby 1994, 2005; Anagnostidis and Komárek 1988), their repression could reduce flotation in the well-lit surface layers, where chytrid infection risk is highest (Reñé et al. 2022). One interpretation is that these changes reflect a host-driven defense strategy. By sinking deeper in the water column, *P. agardhii* may lower its exposure to chytrid zoospores, which are thought to accumulate in highly illuminated water layers (Bruning 1991; Tao et al. 2020; Wierenga et al. 2022). At the same time, upregulation of photosynthetic genes, particularly those linked to phycobilisomes, may represent a compensatory response to maintain energy production under reduced light conditions. In this way, *P. agardhii* could enact a dual strategy: physically escaping high-risk environments while bolstering photosynthetic efficiency to tolerate the lower-light habitat.

Alternatively, changes in photosynthetic gene expression could be chytrid-driven. Increased host photosynthetic activity may benefit the chytrid by enhancing carbohydrate availability during infection and promote the infection of other filaments as it is thought that zoospores may be attracted to photosynthetic by-products (Scholz et al. 2017; Senga et al. 2018). Previous research has suggested that algal chytrids can modulate host metabolic pathways, including the downregulation of those associated with the production of antifungal volatile organic compounds such as β-ionone (Yoneya et al. 2021). Furthermore, chytrid pathogens such as *Batrachochytrium dendrobatidis* are known to manipulate host physiology through the secretion of enzymes, proteins, and metabolites for their benefit (Ellison et al. 2017; Grogan et al. 2018; McDonald et al. 2020; Torres-Sánchez et al. 2022), raising the possibility that similar mechanisms may operate in algal chytrid infections.

Further evidence of a possible host defensive strategy was observed in genes encoding an ABC-type nitrate transporter (Fig. S5). Repression of nitrate uptake and assimilation, which are tightly coupled to photosynthesis (Rai et al. 1981; Flores et al. 2005), could limit nitrogen availability to the parasite. This is consistent with experimental studies showing that chytrid reproduction declines under low N:P conditions (Frenken et al. 2017b). Thus, the observed host transcriptional changes may represent a combination of self-protective responses and chytrid-driven manipulations, underscoring the complexity of host-parasite interactions in cyanobacterial blooms.

Because *R. megarrhizum* chytrids are obligate parasites, we were unable to generate RNAseq controls to directly compare saprotrophic and parasitic states. Instead, we used size-based separation to preliminarily approximate different infection stages for transcriptomic comparison. Across all samples, chytrids showed consistently high expression of cytoskeletal transcripts (tubulin and actin) along with a large proportion of hypothetical proteins (Fig. 3). Successful host infection requires the transition from zoospore to sporangium, a process that depends on complex actin networks (Fritz-Laylin et al. 2017). This transition is also marked by the internalization of cilia and degradation of tubulin (Venard et al. 2020). Consistent with these observations, recent work in *Rhizoclosmatium globosum* demonstrated striking remodeling of microtubule-organizing centers (MTOCs) and centrioles during its coenocytic lifecycle (Long et al. 2025), highlighting dynamic cytoskeletal reorganization as a shared and potentially essential strategy in chytrid development and infection. While this data also suggests that cytoskeleton genes are important in infection dynamics, more work is needed to better associate changes in these gene expression levels with chytrid life stages in this system.

When comparing the hypothetical zoospore fraction to the hypothetical sporangia fraction, we observed increased expression of carbohydrate-binding proteins such as CBD9 and ConA-like lectin/glucanase domain-containing proteins in zoospore fraction, suggesting that carbohydrate recognition may play a key role during the initial stages of host invasion (Fig. S5). Similar mechanisms have been described in other fungal pathogens, where host recognition is mediated by lectin and sugar interactions (Addepalli et al. 2002; Gutman et al. 2011). For example, the lectin concanavalin A (ConA) has been shown to induce zoospore encystment and direct chemotaxis in multiple pathogenic fungi (Hardham and Suzaki 1986; Jansson and Thiman 1992). It has further been proposed that zoospores themselves carry lectins that bind to sugar components of host cells, as host recognition persists even after algal cells are denatured (Hoffman et al. 2008). The prevalence of lectins across fungal lineages, including their abundance in higher fungi (Giollant et al. 1993; Wimmerova et al. 2003), supports the idea that lectin-mediated carbohydrate recognition represents a widespread and conserved strategy for host detection and attachment.

In summary, this study provides the first comparative genomic and transcriptomic framework for chytrids infecting *P. agardhii*, offering new insights into both parasite and host responses during infection. By combining single-cell sequencing, metagenomics, and RNAseq, we generated reference genomes for *R. megarrhizum* and revealed early signatures of cytoskeletal investment and transcriptional shifts that should be further explored during life stage transitions. On the host side, *P. agardhii* exhibited transcriptional trends that were suggestive of altered buoyancy and nutrient acquisition, which may represent defensive strategies or chytrid-driven manipulation. Together, these findings highlight the complexity of chytrid-cyanobacterium interactions and identify cytoskeletal remodeling and carbohydrate recognition as potential markers of infection. More broadly, this work establishes essential genomic resources that will enable deeper exploration of chytrid functional diversity, population structure, host specialization, and eco-evolutionary dynamics within parasitic chytrid systems.

## Supplementary Information

Below is the link to the electronic supplementary material.

Supplementary file1 (XLSX 875 KB)

Supplementary file2 (DOCX 2795 KB)

## Data Availability

Metagenome raw reads and assembled genomes (MAGs) for the chytrid isolates were uploaded to NCBI under Bioproject ID PRJNA1356482. Raw RNA-seq (metatranscriptomes) of chytrid infected *P. agardhii* samples were uploaded to NCBI SRA under Bioproject ID PRJNA1356502. Annotated single cell genomes are available from JGI MycoCosm (Grigoriev et al. 2014) at https://mycocosm.jgi.doe.gov/PlkC2_A_1 and https://mycocosm.jgi.doe.gov/PlkC2_B_1. The single genomes are also available in NCBI GenBank under Bioproject ID PRJNA1080981- PRJNA1080982.
