# Supplementary material for: Single-cell genomics, metagenomics, and transcriptomics of Rhizophydium megarrhizum, an obligate fungal parasite of Planktothrix agardhii
Source: Aquat Ecol. 2026 Jul 22;60(3):92. doi: 10.1007/s10452-026-10329-8 (PMC13391722; doi:10.1007/s10452-026-10329-8)
Supplement: Supplementary file 2 — Supplementary file2 (DOCX 2795 KB) [file 10452_2026_10329_MOESM2_ESM.docx]

Whole Genomes of *Planktothrix agardhii* Obligate Fungal Parasite (*Rhizophydium megarrhizum*) from Single-cell Sequencing and Metagenomics

Supplemental Materials

Table S1. Pangenome overview for the combined single-cell and MAG *Rhizophydium megarrhizum* genomes.

| **Genome** | **# Genes** | **# Genes in Homologs** | **# Genes in Singletons** | **# Homolog Families** |
| --- | --- | --- | --- | --- |
| *R. megarrhizum* C21 | 9913 | 9487 | 426 | 4094 |
| *R. megarrhizum* PlkC2_A | 8572 | 6202 | 2370 | 5877 |
| *R. megarrhizum* C02 | 9988 | 9554 | 434 | 4121 |
| *R. megarrhizum* C24 | 9959 | 9530 | 429 | 4113 |
| *R. megarrhizum* C06 | 9852 | 9435 | 417 | 4082 |
| *R. megarrhizum* C01 | 9845 | 9428 | 417 | 4079 |
| *R. megarrhizum* C22 | 9929 | 9501 | 428 | 4099 |
| *R. megarrhizum* C10 | 9869 | 9449 | 420 | 4086 |
| *R. megarrhizum* C23 | 9819 | 9409 | 410 | 4073 |
| *R. megarrhizum* C03 | 9803 | 9396 | 407 | 4066 |
| *R. megarrhizum* C07 | 9916 | 9490 | 426 | 4096 |

Table S2. Full pangenome ortholog group dataset identified among the *Rhizophydium* *megarrhizum* single-cell isolate PlkC2_A and 10 *Rhizophydium* *megarrhizum* MAGs. Representative genes are shown for each ortholog cluster group across genomes. Ortholog groups are ordered by prevalence across genomes, with clusters at the top shared among all genomes and those toward the bottom unique to a single genome.


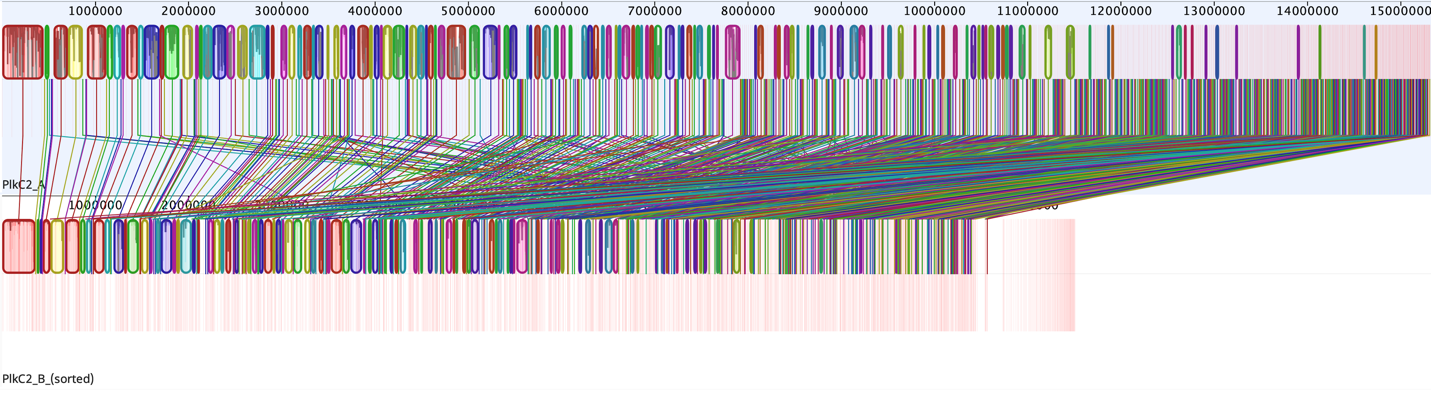


Figure S1. Whole Genome Alignment of *R. megarrhizum* C02 single-cell sequence assemblies (PlkC2_A and PlkC2_B). Colored blocks indicate regions of the genome sequence that aligned to part of another genome and are presumably homologous and internally free from genomic rearrangement. The faint red lines that span the genome indicate contig breaks. Regions with no blocks were not aligned and contain unique sequence elements. These two genomes generate 3965 locally collinear blocks.


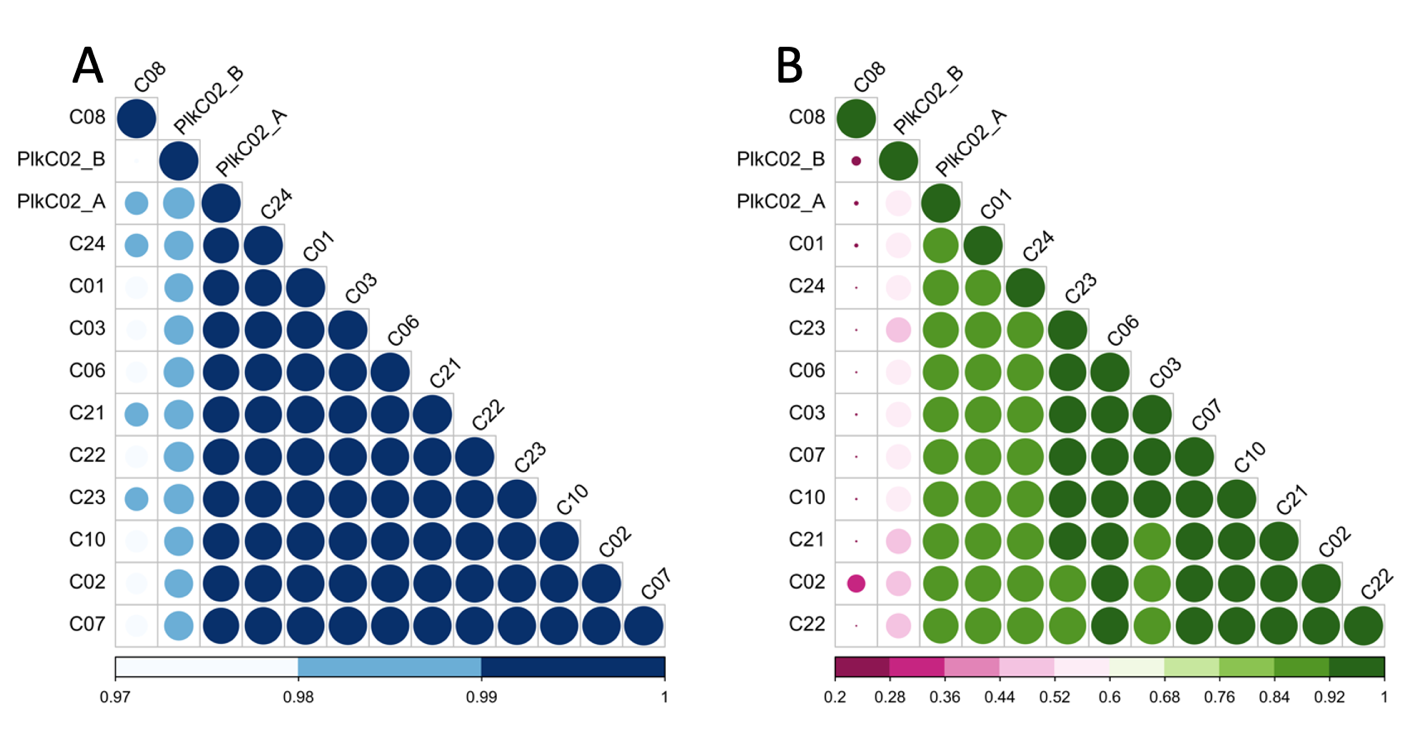


Figure S2. Genomic similarity comparisons among *R. megarrhizum* single-cell sequences and metagenome-assembled genomes (MAGs). Average Nucleotide Identity (ANI; panel A) and Alignment Percentage (AP; panel B) are shown for *R. megarrhizum* single-cell sequences (PLKC02_A and PLKC02_B) and metagenome-assembled genomes (MAGs).


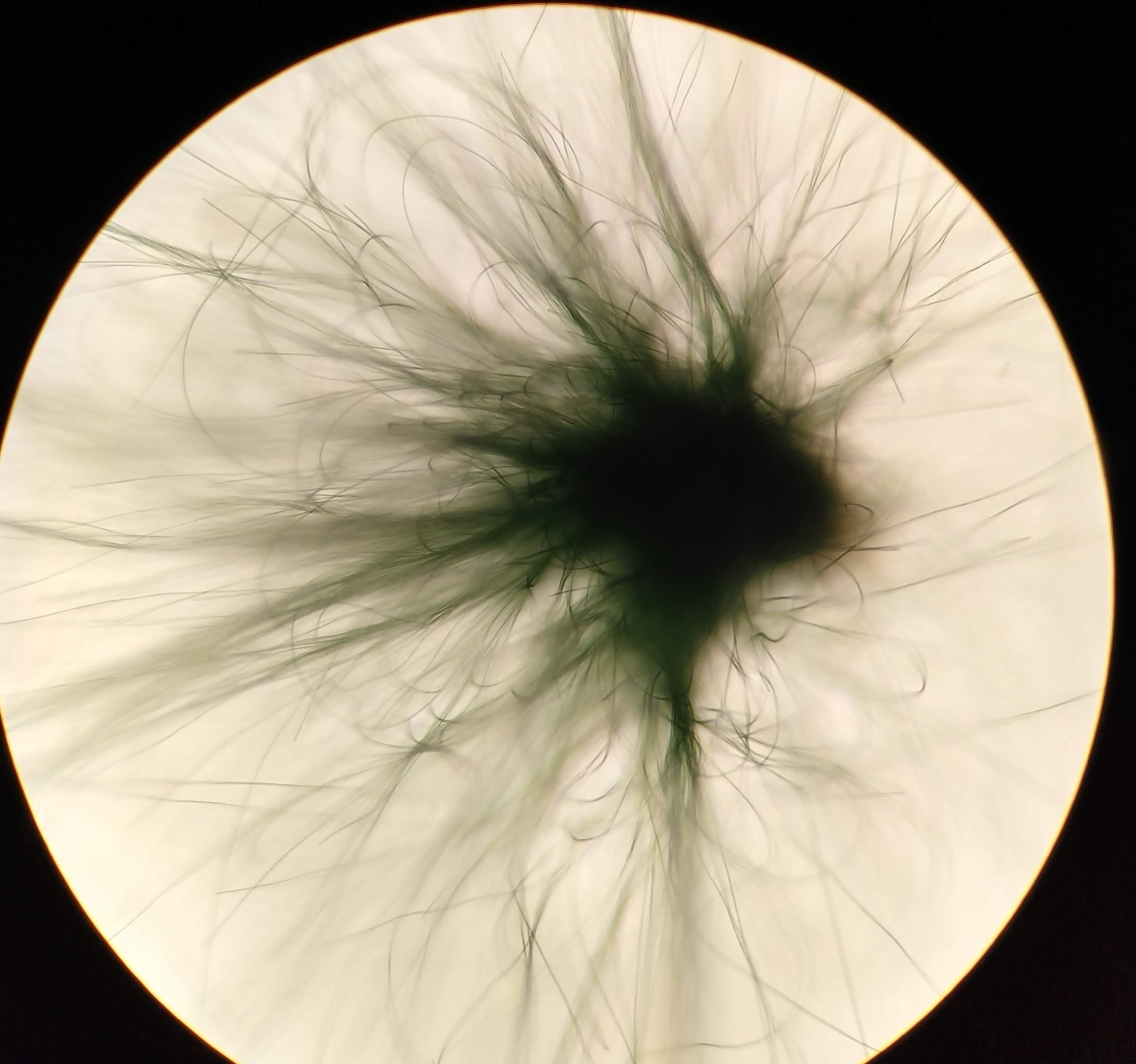


Figure S3. Photo image of ball defense aggregate of C02-chytrid infected *Planktothrix* 1031 taken at 100x magnification.

Figure S4. Taxonomy of the RNAseq trimmed reads.


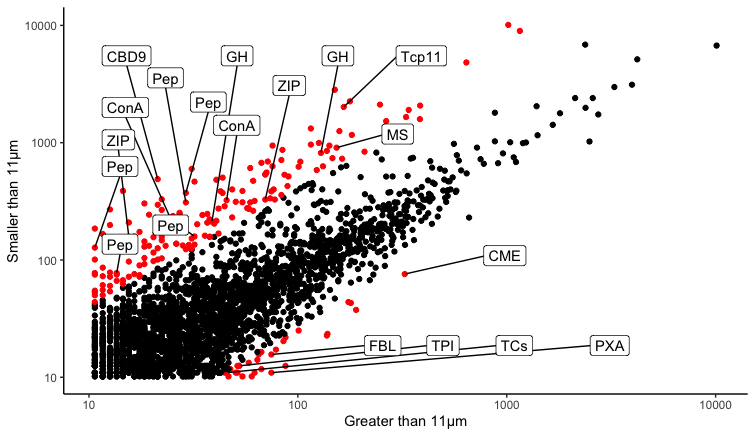


Figure S5. Expression profile comparison between small infective particles (zoospores) and large infective particles (sporangia). Red data points have a greater than 90 % probability of being differentially expressed calculated from the log_2_-ratio of the two conditions. Gene abbreviations include carbohydrate-binding family 9-like protein (CBD9), Muniscin C-terminal μ-homology domain-domain containing protein (CME), Concanavalin A-like lectin/glucanase domain-containing protein (ConA), Fibrillarin (FBL), Glycoside hydrolase (GH), Malate Synthase (MS), Peptidase S8/S53 domain-containing protein (Pep), PXA domain-domain containing protein (PXA), T-complex protein 11-domain containing protein (Tcp11), Terpenoid cyclases/protein prenyltransferase alpha-alpha toroid (TCs), Triosephosphate isomerase (TPI), and Zinc/iron permease (ZIP).

Figure S6. Expression profile comparison between Uninfected *Planktothrix agardhii* 1031 and the chytrid infected Large (>11 µm) fraction. Red data points have a greater than 90 % probability of being differentially expressed calculated from the log_2_-ratio of the two conditions. Gene abbreviations include photosystem II assembly protein Psb34 (Psb34), bicarbonate transporter BicA (BicA), nitrate ABC transporter ATP-binding protein (ntrCD), CmpA/NrtA family ABC transporter substrate-binding protein (CmpA/NrtA), nitrate ABC transporter permease (ntrB), and LysR family transcriptional regulator (LysR).
